# Comments on “Part I: determination of a structure/property transformation mechanism responsible for changes in the point of zero change of anatase titania with decreasing particle size” by M. Leffler, A. Mirich, J. Fee, S. March and S. L. Suib, *RSC Adv.*, 2024, 14, 30543

**DOI:** 10.1039/d5ra01865j

**Published:** 2025-05-23

**Authors:** Johannes Lützenkirchen, Bahram Hosseini Monjezi, Marek Kosmulski

**Affiliations:** a Institute for Nuclear Waste Disposal (INE), Karlsruhe Institute of Technology (KIT) Hermann-von-Helmholtz-Platz 1, Eggenstein-Leopoldshafen 76344 Germany johannes.luetzenkirchen@kit.edu; b Lublin University of Technology Nadbystrzycka 38 Lublin 20618 Poland

## Abstract

The work mentioned in the title of this comment appears to establish a relationship between the point of zero charge of oxide minerals (anatase, goethite, and others) and particle size. While the methods to establish the point of zero charge used in the experimental work of the paper are non-standard and need to be critically assessed, the apparent increase of the point of zero charge with particle size is clearly not a general feature and some literature data observed by standard methods for measuring points of zero charge actually suggest the absence of a universal trend. Since part II of article series (*RSC Adv.*, 2024, 14, 30317) also builds on this apparent relationship, we believe readers should be cautioned about it. The comment discusses the issues in more detail.

## Abstract 2

We summarize evidence on size effects on points of zero charge, showing no consistent trend, and discuss challenges in accurately determining these values.

## Graphical abstract

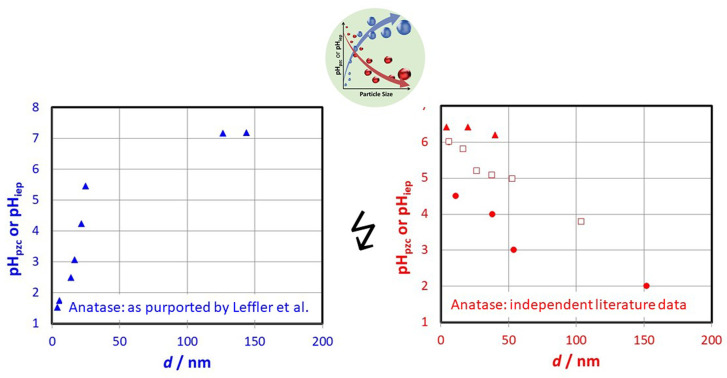

In the first article^1^ in the series of two papers,^1,2^ Leffler *et al.* develop a methodology to relate properties of anatase (and other materials) to particle size. In particular they build their discussion on an increase of the point of zero charge (PZC) with increasing particle size in the small particle range for a given material until reaching a threshold value beyond which the PZC remains constant.^1^

While it might be possible to establish such a relationship for a given material, there is no experimental evidence that PZC will in general increase with increasing particle size at the lower particle size range.

We contend that in the above referenced paper (i) the way the particle sizes are evaluated is debatable and (ii) the way the PZC is experimentally observed and interpreted not only involves a method to determine the PZC, which is questionable, but it also disregards the advances made in understanding the charging of oxide minerals from a molecular scale point of view as related to surface structure. Third, the exclusion of data that are in disagreement with the above sketched trend purported by Leffler *et al.*^1^ appears arbitrary.

Thus, work by Zhou *et al.*,^3^ Ridley *et al.*,^4^ Suttiponparnit *et al.*,^5^ Dumont *et al.*,^6^ or Vayssières^7^ provides numerous examples for anatase and a number of other solids, where the PZC changes in a way opposite to the trend purported by Leffler *et al.* In particular, the work by Ridley *et al.*^4^ and Vayssières^7^ each involves experimental results from one laboratory using consistent methods for particle size determination and PZC determination, in those cases potentiometric titration (PT). The experimental approach is only to some extent comparable to the work of Leffler *et al.*^1^ which they refer to as mass-titration (MT) because the set of PTs by Leffler *et al.* for each solid specimen involves one PT without solid and two more at two solid concentrations, all at one electrolyte concentration. Conventional PTs to determine proton related charge densities and PZCs involve acid–base titrations at different salt concentrations, which are corrected for the blank. If done with the necessary care, the common intersection point (of PT curves), CIP, may be the PZC (see below).^8^ The conventional MT involves the addition of solid to a solution until the pH of the suspension reaches a plateau.^9,10^ This pH may be the PZC. This is explained also in some detail by Bourikas *et al.*^11^, who use an experimental approache to determine a PZC, which is cose to that used by Leffler *et al.*,^1^ but they involved three solid concentrations and do not apply the approach to solids with PZC < 3. In the work by Suttiponparnit *et al.*,^5^ where materials also originate from one laboratory with the same methods applied to the determination of particle size, the PZC is determined *via* electrokinetics. In essence the approach taken by Leffler *et al.*^1^ is not one of the standard approaches. The size determination by Leffler *et al.*^1^ applied to literature data involves, *e.g.* for a group of goethite preparations, the determination of an equivalent size of a sphere for the typically non-spherical goethite particles using the measured specific surface areas. In particular with reference to the role of specific crystal planes this is an oversimplification. Moreover, particle size (monodisperse and spherical particles) and apparent mean particle size in an assembly of polydisperse and irregularly shaped particles, *e.g.* from their specific surface area are two different issues. Such assemblies also will occur for monodisperse, spherical particles in solution, close to the PZC, due to the coagulation of the primary particles. We will therefore include in our discussion data from the literature for spherical particles, where no recalculation of size is required. Unlike what is stated by Leffler *et al.*^1,2^ the various size determinations applied to the particles used by Suttiponparnit *et al.*^5^ appear to yield a quite consistent picture about primary particle sizes. It is established that hydrodynamic radii may differ from those values. For the data set of Suttiponparnit *et al.*^5^ the recalculation of the diameter from specific surface areas as done by Leffler *et al.* for other data sets would have invalidated the trend in Fig. 4 of the paper by Leffler *et al.*^1^

We now discuss literature that deals with the effect of size on PZC and charging of oxide minerals. Leffler *et al.* for example support their opinion by citing the paper by Barisik *et al.*^12^ stating “They (*i.e.* Barisik *et al.*) observed that as the surface area increased (*i.e.* decreasing particle size) PZC values were found to increase”.^1^ However, Barisik *et al.*^12^ just report a surface charge dependence of silica as a function of size. Those authors retrieved a trend on which common agreement prevails in the community: below a certain threshold (that depends on ionic strength and charge density) the size of spherical particles starts to affect the basic charge density due to protonation/deprotonation of surface hydroxyls and charge density increases with decreasing size (for spherical, homogeneously charged silica particles). The change in charge density with particle size is caused by the curvature of *e.g.* spherical silica particles, which at a certain particle size (depending on pH and background electrolyte concentration), starts to affect the electric field (and thus the shielding of charge) and ultimately allows more sites per surface area (in the case of silica) to deprotonate for the smaller sizes. Once the particles become sufficiently large, this effect disappears and the surface charge densities overlap for those sizes and the system can be modelled as if it were a planar surface. In the context of the present discussion it is important to note that the paper by Barisik *et al.* does not discuss PZCs at all (the term is not even mentioned once in the paper). Instead, the graphs that would allow a guess of PZCs indicate that charging curves for various sizes coincide at low pH, where the surface charge density trends to zero. The PZC of these kinds of silicas is ill-defined due to this plateau and the absence of positive charge. Despite these problems some authors discuss experimental PZCs of spherical silica particles^13^ and obtain decreasing PZCs with increasing size, but due to the problems with the very low zeta-potentials close to the PZC, the rather flat curves and the associated experimental errors, we believe that the trend is not reliable in those cases. Others discuss a decrease of the PZC of silica with increasing particle size.^14^ Since for silica, it has been reported that the site-density changes with particle size,^15^ size-dependence has to be studied systematically by using the same kind of particles, with the same site density, such that only particle size is varied. For the small sizes, probably it will also be required to take into account the expected size-dependence of solubility.^16^ As small particles show higher solubility the concentration of solution monomers will be higher for dispersions of small particles, and the contribution of solution monomers to the proton balance will be higher. This will affect the apparent surface charge determined by titration. The problem is general and it applies to all kinds of fine particles irrespective of their chemical nature. So mainly the paper referenced by Leffler *et al.*^1^ deals with surface charge densities as do others for non-spherical particles.^17^ In the latter paper, there is no significant effect of particle size on PZCs in most of the figures for goethite. However, Abbas *et al.*^17^ state “The CDH-SC theory also predicts that the pH_pzc_ value shifts toward higher pH values as the particle size decreases”, *i.e.* opposite to what Leffler *et al.* show in their graphs. This opposite trend is reported a few years later for TiO_2_.^18^ In summary, the paper by Barisik *et al.* clearly does not provide any support for a change of the PZC with size. Changes in surface charge density with size for a given family of particles does not have a causal link to PZC. The above quotation from the paper by Leffler *et al.*^1^ unfortunately mixes up the two issues.

In the most advanced literature on the PZC of oxide particles, the role of the exposed crystal planes has emerged as the important factor. Leffler *et al.*^1^ in their list of factors influencing PZCs have included morphology and roughness with reference to Borghi *et al.*^19^ The most well-known example is the plate-like form of clay minerals with typically permanent (negative, largely pH-independent) charge on basal planes and pH-dependent charge on the edge planes. For oxide particles modern site-binding (surface complexation) and molecular scale based models suggest that the PZC can be varied by modifying the relative contributions of different crystal planes. Thus the PZC of goethite crystal planes was found to vary between 3.8 for the (021) face, 7.8 for the (010) face, and 8.5 for the (110) face for ideal, defect free crystal planes as recently reported based on quantum chemistry.^20^ Another theoretical study has more recently appeared for titania using a somewhat different method to evaluate the PZC.^21^ For rutile, the face specific PZCs reported were 4.28 (011) and 5.39 (110), and those for anatase were 5.88 (001), 5.95 (101) and 7.02 (100). The general idea stems from the fact that different surface oxygen atoms will have different proton affinities and different crystal planes will expose different surface oxygen atoms or different combinations of those surface oxygen atoms. This outcome from quantum chemistry is in good agreement with semi-empirical surface complexation models such as the MUSIC model that simulate experimental titration data for example with such a concept.^22^ Experimental evidence of face-specific charging is also available for α-Al_2_O_3_,^23^ rutile,^24^ or SrTiO_3_.^25^ For rutile (110) the experimental IEP was reported to be between 4.8 and 5.5,^24^ and the one based on quantum chemical calculations is 5.39,^21^ while another experimental study reports a value of 4.8 ± 0.3.^26^ Yet another “theoretical” value from a combination of the MUSIC model and quantum chemistry is 4.76.^26^ These values are remarkably close and all close to or within the range 4.8 and 5.5. For rutile (100) we found two experimental values in the literature (3.2–3.7 from Bullard *et al.*^24^ and 3.47 from Borghi *et al.*^19^). These values are clearly lower than those reported for the (110) face, which clearly supports the idea of a face specific PZC. In real systems this will to some extent be affected by defects, and this has been studied in great detail for goethite,^27,28^ but does not fundamentally affect the evidence that different faces of a given mineral will in general have different PZCs. The relative contributions of the faces and their geometrical arrangement will ultimately determine the overall PZC of a particle of certain habit and “size”. The two above factors may change with particle size and result in changes in PZC. Tailoring particles in terms of morphology and size thus will ultimately allow to generate particles of target sizes with target PZC. For very small particles, the morphological approach becomes difficult if not impossible and even changes in site density with size have been reported.^15^ As a consequence, understanding the charging will for a given sample require determining the nature of the exposed surface functional groups and their respective quantities as has been done for ferrihydrite for example.^29–32^ In principle, tools are available to accomplish this. Even from this view angle it is possible to tailor the surface chemistry and thus the PZC for a given size. A factor that may be complicating this, are potential changes in interfacial water structure, which could affect hydrogen bonding.

In summary, in general, a universal size dependence as is purported in the paper by Leffler *et al.* is not expected.

Fig. 1 shows a collection of PZCs as a function of size for anatase and other solids. We are not discussing the way of PZC determination here, since the methods used in the referenced articles are accepted standards while the approach taken by Leffler *et al.*,^1^ is not. From Fig. 1a it becomes clear that none of the literature data sets that we plot for anatase (red symbols) corroborates the trend purported by Leffler *et al.*^1^ (black symbols). The work on anatase by Ridley *et al.*^4^ shows nearly no dependence, notably at the low particle sizes (full red circles). The data point from Ridley *et al.* at 5 nm (open red circle) is based on estimates of the size from specific surface area measurements and involves a coinciding value (PPZC = 6.85) from electrophoretic mobility and potentiometric titrations^33^ that is even higher than the value (PZC = 6.42) for the 4 nm sample from their later studies, which is based on CIP.^4,35^ The difference between the two values is related to the interpretation of the titration data, but in essence, both values are sufficiently high to clearly contradict the universal trend hypothesis. Data by Zhou *et al.*^3^ also show a decrease of reported PZC with increasing site density as do the results of Suttiponparnit *et al.*,^5^ which have been discussed earlier, and will be later again.

**Fig. 1:**
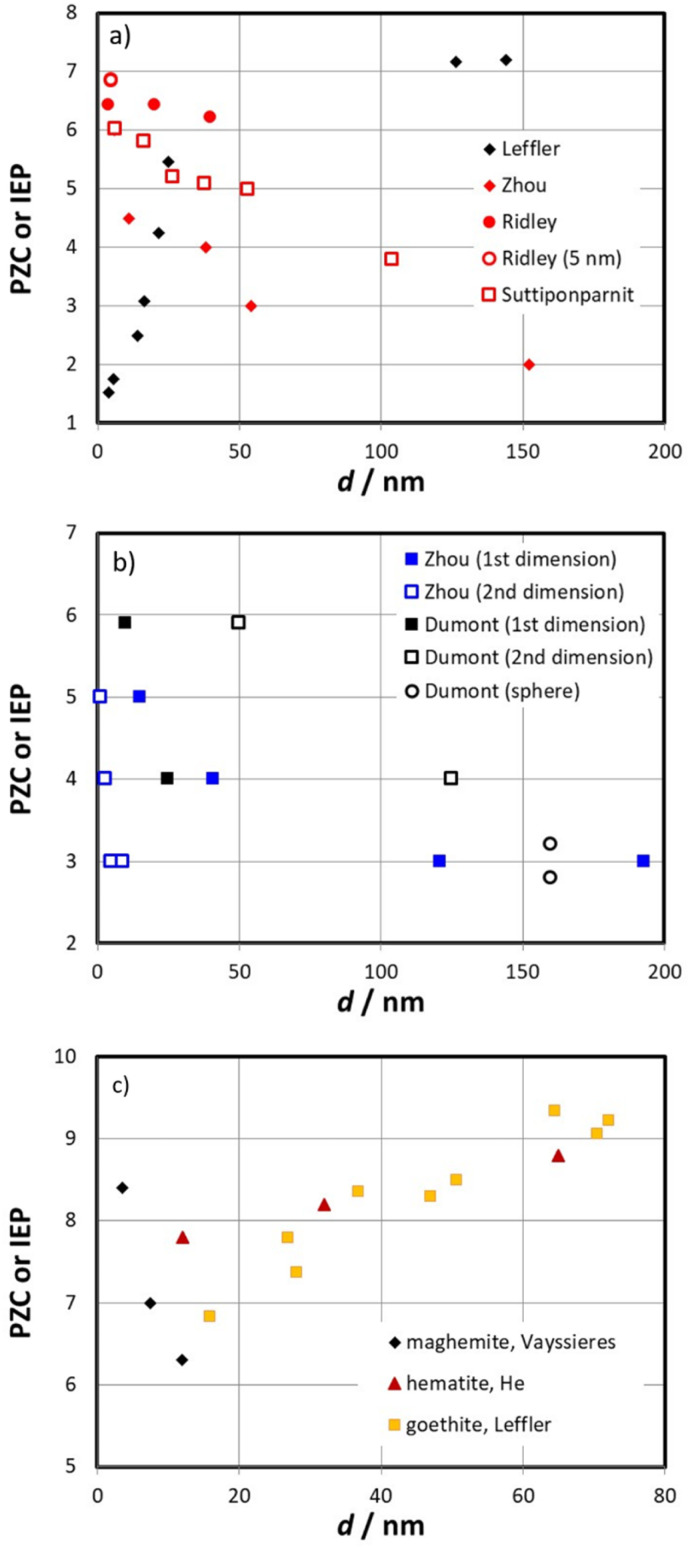
PZCs as a function of particle size. (a) Literature data for anatase from Zhou *et al.*,^3^ Ridley *et al.*,^4,33^ and Suttiponparnit *et al.*^5^ and those of Leffler *et al.*^1^ (b) Literature data for rutile rods from Zhou *et al.*,^3^ acicular rutile from Dumont *et al.*,^6^ and rutile spheres from Dumont *et al.*^6^ (c) Literature data for iron(iii) oxides: maghemite from Vayssières,^7^ He *et al.* for hematite,^34^ and those for goethite used by Leffler *et al.*^1^ PZC/IEP determinations: Zhou: IEP, Ridley:^4^ CIP, Ridley:^33^ CIP, IEP, Suttiponparnit:^5^ IEP, Dumont:^6^ stability, Vayssieres:^7^ CIP, He:^34^ IEP.

Fig. 1b shows literature data for rod-like/acicular rutile particles. Independent of the dimension chosen for these non-spherical particles, the trend is opposite to that purported by Leffler *et al.*^1^ Moreover, the data point for the 160 nm spherical particles from Dumont *et al.* with a PZC range between 2.8 and 3.2 (the range indicates the uncertainty reported by the original authors) shows that even for rather large monodisperse particles, low PZCs have been reported. We emphasize that the rutile samples used by Dumont *et al.* were carefully cleaned prior to the experiments.^6^ They determined the PZC using aggregation experiments in a range of electrolytes.^6^ Despite the small number of points, the data are from one laboratory and in disagreement with a universal trend of PZCs.

Fig. 1c finally includes some results for iron(iii) minerals. While the hematite data show the same trend as the data set for goethite, the behaviour of maghemite is opposite.

To sum up, we can find any kind of trend of PZC with particle size at the small size range for a given solid (increasing, decreasing, none at all).

To obtain an unambiguous set of data for PZC as a function of particle size, one should use particles obtained in an identical way (*i.e.* with the same structure, morphology *etc.*) that just vary in size, while keeping all other properties constant including *e.g.* site density. This is quite difficult and requires a lot of effort. After going through the previous studies (all from the same laboratory or with the same source of particles) related to the paper of Suttiponparnit *et al.*^5^ we conclude that the particles prepared for the paper by Suttiponparnit *et al.* for particles of 6, 16, 26, 38, 53, and 104 nm are probably such a rare example.^5^ Unlike the statement by Leffler *et al.* that this work shows “how difficult it is to determine an accurate primary particle size”, the references provided by Suttiponparnit *et al.* report in detail the synthesis and characterization of those particles. Notably the primary particle size of those particles has been very well studied.^36–39^ From our point of view, there is no reason to exclude from the discussion the results for these particles. On the contrary, they seem to be rather well characterized compared to many other studies. Clearly, this data-set shows the opposite trend of PZC with particle size compared to that purported by Leffler *et al.*

We may also refer to the work by Suttiponparnit *et al.*^5^ again to discuss in more detail the determination of PZCs for oxide minerals in more detail. There is little doubt that in an inert electrolyte, the pH at which the oxide is uncharged corresponds to the PZC. Charge can be determined in a number of ways. The simplest and most unambiguous way is *via* electrokinetics in suspensions of low ionic strength when taking all required precautions. This in the case of nanoparticles typically involves commercial setups such as the Malvern, Brookhaven or Anton Paar devices for example. Suttiponparnit *et al.*^5^ for example used 1 mM NaCl solutions for their experiments with a Malvern device (we note here that Suttiponparnit *et al.*^5^ do not state that they worked in the absence of carbon dioxide, see discussion below). Electrokinetics at low salt concentrations, *i.e.* in the absence of background electrolyte or ≤1 mM monovalent salt such as KCl, NaCl or the like yields an isoelectric point (IEP). Another established way is the use of electroacoustics.^40^ It has the advantage to involve high solid concentrations, whereas the low solid concentrations in other methods make the measurement susceptible to contamination. At the low concentration of supposedly inert salt the IEP can be assumed to be close to or identical to the real, pristine point of zero charge (PPZC) of the sample, since even if a component of the salt were to be specifically interacting, the effect would be minimized at low concentrations. Therefore, such an IEP can be taken as the PPZC, which for oxide-type minerals represents the PZC unaffected by any of the solution components except protons and hydroxide ions. Doing an electrokinetic experiment on a given sample at two different electrolyte concentrations of the same salt for example also yields the PPZC if the IEPs coincide. Potentiometric titrations have also been used to determine PZCs. There is agreement that these kinds of acid–base titrations only yield relative surface charges with respect to an unknown initial state of the suspension. Doing at least three titrations (*i.e.* identical procedure but in three salt concentrations of the same salt) from an identical initial state of a suspension (*i.e.* a stock suspension) in a carbon dioxide free environment, ideally leads to a CIP. If the salt is not affecting the PZC (*i.e.* the components of the salt are inert, both the cation and the anion of the background electrolyte are non-specifically adsorbing, or both cation and anion have exactly the same affinity), the CIP is the PPZC. Unfortunately, a CIP may also be found in electrolytes containing specifically adsorbing ions.^41^ As a consequence, one has to prove that the background salt does not shift the PZC. By changing the salt (*i.e.* NaCl *vs.* KCl *vs.* NaNO_3_), it becomes possible to find the PPZC, but this involves very careful and tedious work. Thus, the CIP is best supported by an IEP to make it qualify for a safe PPZC. This has been known for a long time. Another way of determining the PPZC is *via* a mass titration, where solid is added to a solution until the pH does not change with the mass of solid any longer.^9^ For this to be valid the solid must be free of impurities, meaning it typically has to be washed to remove impurities.^10^ The method of Leffler *et al.* from our point of view does not qualify at all for PZC determination. In the data we use in Fig. 1 and in the main text, we have PZCs determined *via* electrokinetics,^5^ colloid stability,^6^ force distance curves,^24^ potentiometric titrations,^4,7^ molecular dynamics,^19,20^ that is, methods different from that used by Leffler *et al.*^1^ For the classical MT type context, the solid concentrations have to be sufficiently high and there is probably not sufficient variation in the work by Leffler *et al.*^1^ Actually it is the exposed surface area that is relevant, so for high surface area (small) particles lower particle concentrations will be required to reach the PZC. Compared to classical potentiometric titrations, the variation in ionic strength is missing. What is more, acid–base titrations become difficult at extreme pH values, since the amount of acid required to decrease the pH is often much higher than the amount related to reactions with the surface. It is a question of exposed surface area, but experience shows that below pH 3 the surface charge density is a small number calculated as a difference between 2 large and almost equal numbers, and this always results in a large relative error, even the sign of the difference can become uncertain.

Leffler *et al.*^1^ claim that addition of 0.01 M acid to titania dispersions can induce pH 1.6. This is impossible. The authors even cite three studies (ref. 42–44) in support of their data. However the cited references do not state explicitly that someone added dilute acid to a titania dispersion and obtained more concentrated acid. Second, the same happens in the absence of titania in the work by Leffler *et al.*,^1^ see their Fig. 22. We also draw attention to the order of the three titration curves, *i.e.* at high, low and zero solid concentration. Considering the data in Fig. 22–24 in the paper by Leffler *et al.*^1^ and assuming that the common intersection point is the PZC, we derive from the data that more titrant has to be added below the PZC for the blank titration to decrease the pH to the same value than is necessary in the presence of the solids. However, below the PZC there is net uptake of protons on the surface (the surface assumes more and more positive charge, and it is the intent of performing acid–base titrations to just figure this out), so that some protons added should go to the surface and thus cannot decrease the pH. Thus, the data are entirely contrary to the expectation and to the observations in hundreds of articles. To us this just confirms that something must be wrong with the data. The results in Fig. 25–27 on the other hand show the expected trends, in our view, simply because the experiments were done in moderate pH ranges (covered by the applied calibration range), where significant amounts of the titrant added go to the surface relative to what is needed to change the pH. Unfortunately, the PZCs at the low pH end are the relevant ones in this study, and they are the least reliable.

The problem has a solution without far-fetched assumptions like water splitting.

(1) The pH electrodes are best suited for measurements at moderate pH values (3–11) and the response beyond this range is not necessarily linear. Actually, linearity should be checked in any pH-range studied.

(2) The pH electrode is typically calibrated with pH buffers of pH 4–10 (in the present case^1^ by three buffers at pH 4, 7, and 10). The experimental error in measuring pH is moderate in the pH range covered by the buffers, but it can be (and apparently in the study by Leffler *et al.*^1^ it was) substantial outside that range.

Finally, while Leffler *et al.*^1^ took precautions to avoid silica contamination, we would like to point out that both in the main text and in appendix D of the paper by Leffler *et al.*^1^ no statement can be found about the gas atmosphere in the titration experiments. If ambient atmosphere was allowed, the systems have been in contact with carbon dioxide. Carbon dioxide may also be present in the sodium hydroxide solution used in some of the titrations. It is well established that the presence of aqueous carbonate species can affect the PZC of aluminium and iron(iii) oxide type minerals.^45,46^ While we did not find a dedicated published study concerning the relevance of CO_2_ to the charging of anatase or TiO_2_, it would seem important to rule out the possibility that CO_2_ affects the PZC of titania type minerals; however, we do not expect that carbon dioxide will have no effect on titania PZC. We already mentioned that in the work by Suttiponparnit *et al.*^5^ the atmosphere during the electrokinetic measurement has not been mentioned, so we have the same kind of uncertainty as with the data by Leffler *et al.* in that respect. This would not only be a problem in the determination for PZCs that occur at higher pH, since CO_2_ has been recently shown to affect charges of solids in aqueous solutions in a more general way and even on surfaces where it has often been assumed to not adsorb.^47,48^ So for example while the presence of CO_2_ can in principle have affected PZCs at the larger particles sizes obtained (Fig. 4 of Leffler *et al.*^1^) directly by forming surface complexes, at the low pH it may have interfered in the titration curves indirectly. For a consistent evaluation of the effect size has on interfacial properties, it is necessary to have particles of the same kind at different sizes and eliminate all (known) interferences, like CO_2_, silica dissolution from glassware, contamination of the original particles *etc.* We do not claim that the literature data we have discussed fulfil these requirements as is not the case for the paper by Leffler *et al.*^1^

For sure one of the goethite data points with low PZC in Fig. 1 of Leffler *et al.*^1^ can be attributed to their ref. 53. In that paper Herrera and McBride^49^ write “the pH values at which the most flocculation was observed were between 7.0 and 7.7, suggesting a PZC in this range. Although this is a lower PZC than reported for goethite based upon potentiometric titration, it is probably shifted to lower pH by the presence of adsorbed HCO_3_^−^…, since no attempt was made to exclude CO_2_ from the suspensions”. We note that inclusion of this PZC in Fig. 1 of Leffler *et al.* may be debatable.

To finish our discussion we would like to focus on hematite systems also in relation to the data plotted in Fig. 9 of Leffler *et al.*^1^ We attempted to disentangle the choice of data for hematite. We only give one example. With respect to the cited ref. 88,^50^ the PZC (7.7) and size (55 nm) for hematite given in that paper is actually from an earlier paper^51^ involving the same hematite, where two PZCs had been reported that appear to have been inferred from potentiometric titrations (7.2 and 7.8). The original data are not given. Pursuing the literature search to that paper, which could have contained the original data,^52^ it turns out that they cannot be found but that electrokinetic data were obtained on the 55 nm sample. The experimental conditions for these do not include a statement on the absence of CO_2_, although such a statement can be found in the section on titrations. So, it might be the safer alternative to exclude this data set. The raw data of the titrations are probably included in a thesis, which we could not access. The IEP of the sample after direct measurement (*i.e.* short equilibration, which could amongst other reasons be linked to limited CO_2_ interference, but we clearly speculate here) is 9.5. Overall, this data set would not pass a critical evaluation for reliable PZCs. Interestingly, in ref. 88 cited by Leffler *et al.*, the authors^50^ compare their commercial sample to one of the synthetic ones of Vermeer.^53^ This sample B is nearly monodisperse, spherical with a size of 50 nm and a PZC of 8.9, which would add a point with high PZC at relatively low size in the hematite plot in Fig. 9 of Leffler *et al.*^1^ as do the hematite data in Fig. 1c. Obviously, there is a plethora of results that could be added to the discussion, or excluded from it. We contend that even this short discussion on hematite precludes the uniform size dependence in the paper by Leffler *et al.*^1^

## Conclusions

To sum, while it cannot be excluded that the low PZCs reported for the smallest particles by Leffler *et al.*^1^ could be reproduced by more appropriate methods, we contend that they are far too unreliable to be used in the articles by Leffler *et al.* including the second part.^2^ Taken together with the evidence that we report from the literature that there is no universal trend of PZCs with particle size based on experiments, we believe that the over-interpretation in the series of papers by Leffler *et al.*^1,2^ will lead to confusion. We have also provided literature evidence that a universal trend is not to be expected, but that overall PZC of an oxide particle is a complex function of the exposed surface hydroxyl groups, and in particular for particles with well-defined morphology for example the relative contributions of crystal planes will determine the overall PZC.

## Data availability

There is no data that could be made available in this comment.

## Author contributions

All authors contributed equally.

## Conflicts of interest

The authors declare no conflicts of interest.
